# Separated and reunified: An apparent time investigation of the voice quality differences between Hong Kong Cantonese and Guangzhou Cantonese

**DOI:** 10.1371/journal.pone.0293058

**Published:** 2023-10-18

**Authors:** Roxana S. Y. Fung, Eugene Y. C. Wong

**Affiliations:** 1 Department of Chinese and Bilingual Studies, The Hong Kong Polytechnic University, Hung Hom, Hong Kong; 2 Department of Speech-Language-Hearing Sciences, University of Minnesota, Minneapolis, Minnesota, United States of America; Education University of Hong Kong, HONG KONG

## Abstract

Hong Kong Cantonese (HKC) and Guangzhou Cantonese (GZC) are two major accents of Cantonese spoken in two geographically non-contiguous cities in Southern China. Previous studies were unable to identify the phonetic features that discern the two accents since they share the same phonological system. This study attempted to solve the puzzle by investigating the voice quality differences between the two accents through acoustic analysis on the speech output of 191 talkers in three age groups ranging from 18 to 65 years old. Among the various spectral and noise measurements of voice quality, we found that Cepstral Peak Prominence (CPP) was the best acoustic measure to discern the two accents. Based on the CPP measure, GZC had overall increased noise than HKC. Covariation of voice quality and tones was studied. The greatest CPP differences between the two accents were found in the two extreme tones: the high-level and the extra-low-level tones. Furthermore, creaky voice was found mainly tied to the extra-low-level tone in both accents. However, HKC exhibited higher frequency of creaky voice than GZC. The creaky voice in GZC was characterized by increased noise and increased tension, compared to those of HKC. Finally, age was found to be a mediating factor in the voice quality of the two accents. Adopting the Apparent Time Framework, voice quality in the two cities has undergone changes over time. The voice quality of the young generations of the two accents have become merged among the three low tones. Furthermore, the prevalence of creaky voice was increasing across age groups in both accents, and it increased at a faster rate in HKC than GZC.

## Introduction

Hong Kong Cantonese (HKC) and Guangzhou Cantonese (GZC) are two prestige accents of the Yue dialects in China. HKC is spoken in Hong Kong, a former British colony; whereas GZC is spoken in Guangzhou, the capital of Guangdong province in southern China. Unlike other English dialects, such as American English and British English, which have noticeable differences in their segmental sounds, the two Cantonese accents are highly similar in their sound systems, except for the realization of the high-level tone (T1) in their six contrastive tones. However, native speakers have no problem in distinguishing two accents without the presence of T1. Previous studies fail to identity the phonetic features that discern the two accents. We attempt to solve this puzzle from a less-studied phonetic domain—voice quality (VQ). The introduction section is structured as follows: We first present an overview of the evolution of the two Cantonese accents and the tonal system of Cantonese. We then proceed to review different phonation types and their association of lexical tones in Cantonese. Finally, we present our research goals.

### Hong Kong Cantonese and Guangzhou Cantonese

Although Hong Kong and Guangzhou share the same principal language, the two cities are geographically non-contiguous. The two cities are 131km (82 miles) apart, separated by other varieties of Yue dialects which are substantially different from HKC and GZC. Before 1842, GZC and the dialect spoken by the native inhabitants of Hong Kong were nearly mutually unintelligible. Based on previous studies on the historical development of Cantonese (such as [1, 2]), we propose that GZC and HKC have been undergoing three different stages due to population movement after 1842, when the British acquired Hong Kong as their colony.

#### Stage I: The *koinized* stage

After 1842, a large number of workers and merchants from the Guangdong province migrated to Hong Kong in wake of the clan wars and civil wars in mainland China. GZC, the lingua franca of Yue dialects, then replaced the local dialect as the dominant language in Hong Kong. At this stage, HKC should be very similar to GZC.

#### Stage II: The *separated* stage

The hundred years of connection between the two cities was blocked around 1950s because of the political movements in China. During this period of separation, the two cities were under different socio-political systems and their languages were subjected to different forces of change. The two accents may have started to develop their own linguistic features.

#### Stage III: The *reunified* stage

The two cities were reconnected in the 80s after China adopted the Reformation and Open-Door policy. Due to the impact of population movement from China to Hong Kong and the propagation of Hong Kong pop-culture to Guangdong province, the linguistic differences between the two Cantonese accents were likely diminishing. In short, HKC and GZC have undergone the *koinized*, the *separated* and the *reunified* stages.

Given the historical development of the two accents, one might expect synchronic variations in their sound systems as a result of the diachronic changes. However, previous works on the phonetic differences of the two accents are limited. Among the published works, most of them have small sample sizes and were unable to reach a conclusive result. For instance, Lee [3] compared the duration and position of the vowels from three HKC and three GZC talkers. It was found that the long vowels /i, y, ɛ/ were more front in HKC than GZC, and the short vowels /ɐ, ɵ, ʊ/ were shorter in duration for HKC. Lee’s observed differences were not replicated in a latter study by Wu [4], in which the consonants, vowels and tones produced by ten HKC and ten GZC undergraduate students were analyzed. The major outcome of Wu’s acoustic analysis showed that GZC speakers had a higher tongue position when producing some vowels compared to HKC speakers. However, Wu argued that the differences in vowels between HKC and GZC were subtle, and that the most distinctive phonetic feature was found in the tonal inventories (the high-falling variant of the high-level tone in GZC). Another study by Leung [5] also found that the high-falling pitch contour was the most noticeable feature in differentiating GZC from HKC, yet this variant is diminishing among the young talkers. It seems that the most significant phonetic difference of the two Cantonese accents lies in their tones. A detailed description of the Cantonese tones should be in order.

### The tonal system of Cantonese

**Fig 1:**
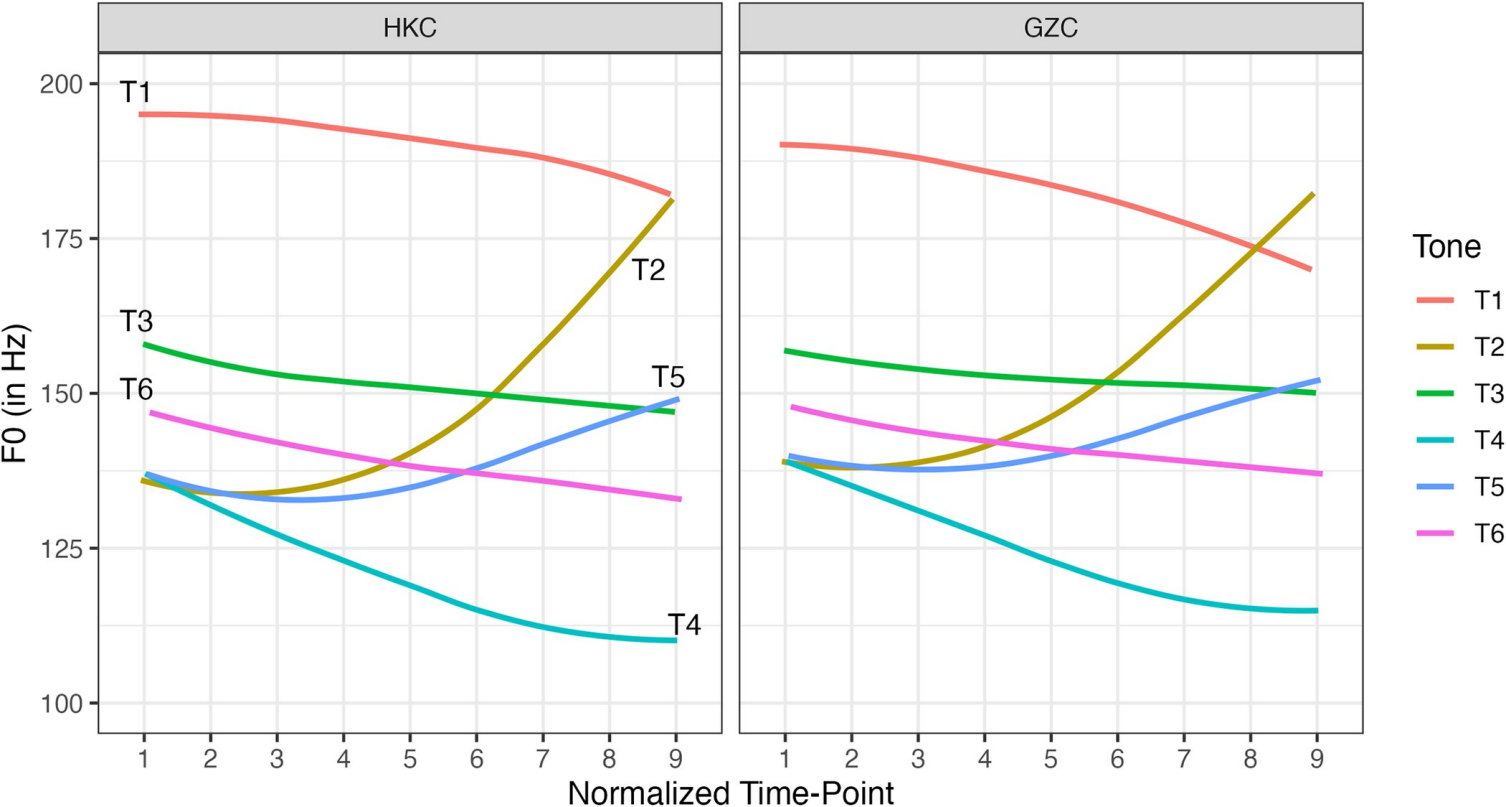
F0 trajectories of the six contrastive tones in Hong Kong Cantonese (HKC) and Guangzhou Cantonese (GZC).

Cantonese is renowned for having a rich tonal system. Table 1 shows the tonal categories and the tone values of the six contrastive tones in Cantonese using Chao tone numerals. The Chao tone numerals indicate the tone height on a five-point scale, with 1 representing the lowest pitch and 5 the highest pitch relative to the talker’s pitch range. Fig 1 displays the f0 trajectories of the six tones in HKC (left panel) and in GZC (right panel), pooled across all talkers in the current study. The tonal system is comprised of four level tones: high-level (T1), mid-level (T3), low-level (T6) and extra-low-level (T4); and two rising tones: high-rising (T2) and low-rising (T5). It should be noted that a falling variant may be produced in the two extreme tones, the high-level tone (T1) and the extra-low-level tone (T4). As reported in previous studies, such as [4–6], the high-falling contour of T1 is attested in some GZC speakers, typically among the older generation. T4 may be realized with a low-falling contour in both HKC and GZC. Moreover, the tonal system of Cantonese is highly symmetrical not only from a synchronic viewpoint but also from a diachronic perspective. Historically, the three low tones (T4, T5 and T6) were split from the three non-low tones (T1, T2, and T3) respectively after the devoicing of the initial consonants in the 9^th^ century AD [7, 8].

**Table 1:** Tonal inventory of Cantonese.

Tonal category	Tone values
T1 (High-level)	55/ 53
T2 (High-rising)	35
T3 (Mid-level)	33
T4 (Extra-low-level)	22/ 21
T5 (Low-rising)	23
T6 (Low-level)	22

Although the high-falling variant of T1 is a distinctive feature of GZC among some speakers, some native speakers claim that they have no problem in distinguishing the two accents even without the presence of the high-falling pitch contour. Since traditional Chinese dialectology mainly focused on describing phones and tones, the question of which phonetic features make the two accents discernible, particularly in the absence of the T1 variant, has perplexed many Cantonese minds.

### Voice quality and phonation types

In the West, a sizeable amount of research has shown that VQ is an important phonetic descriptor of accents, especially in different social or ethnic varieties of English (e.g. Henton and Bladon [9], Esling [10], Szakay [11]). According to Esling and colleagues [12], there were two main senses of VQ. In the broad sense, VQ refers to the long-term settings of the articulators, which contribute to the long-term characteristics of an individual’s voice. In the narrow sense, VQ refers to the different types of phonation produced at the glottis. Esling and colleagues further pointed out that these two senses are highly interrelated as phonation produced at the glottis is the first element that shapes one’s speech. VQ is the most persistent component of an accent, compared to the other components, such as segments, prosody, and intonation. Even within a short portion of speech, VQ characteristics can be perceived because the voice signals produced at the glottis are present whenever voiced phones are produced (i.e., vowels and voiced consonants). VQ is partly determined by biological make-up; it may reflect the health of the vocal tract, but it is also socially acquired by members of the same linguistic community in a given generation. In short, VQ characteristics can reflect the accent of an entire sociolinguistic group, with individual’s accent varying within the boundaries of the social norms [13, 14].

Traditionally, phonation types have been described as a continuum of different glottal states ranging from voiceless, through breathy voice, to regular modal voicing, and then creaky voice to glottal closure [15]. Modal voice is usually the regular speaking voice produced with vocal folds held together with enough tension to allow periodic vibrations. When the vocal folds are not so constricted and allow significant amount of air escaping through the incomplete closure of the glottis, this phonation type produced is called breathy voice. When the tension of the vocal folds increase and the vocal folds are shortened, creaky voice is produced. However, this continuum model describes only three phonation types. As the vocal folds can be manipulated in different ways, numerous other types of phonation, such as whispery voice, harsh voice, and falsetto voice, can also be produced. Even within one major phonation type, there may be subtypes with distinctive acoustic and auditory qualities. For instance, Keating and colleagues [16] proposed six different kinds of creaky voice, such as prototypical creaky voice, vocal fry and multiply pulsed. These subtypes of creaky voice may involve different articulatory mechanisms and each of them have to be characterized by different acoustic measures. Unfortunately, there are no detailed studies examining the articulatory, acoustics and perceptual relationships of these creaky voice subtypes.

Despite the vast amount of research in English languages, sociophonetic studies of VQ or phonation on Cantonese are scarce. Fung and Lee [17] investigated the social indexing function of VQ in HKC based on the speech output of 60 HKC speakers of three age groups. Results show that the higher-educated senior male speakers speak with a breathier voice than lower-educated peers; whereas higher-educated female speakers speak with a very low (male-like) pitch floor, showing a creaky voice quality. Another study by Fung [18] was the first attempt to explore the VQ setting in discerning HKC and GZC. In that preliminary study, acoustic analyses were administered to the speech outputs of 24 HKC and GZC talkers with age ranging from 20 to 58 years. It was found that the pitch level of HKC was comparable to that of GZC, but HKC had a wider pitch range than GZC. Among the six acoustic parameters used to gauge the VQ of the two accents, all pointed towards that HKC was less breathy than GZC, except H1*-H2*, which suggested the opposite. The results were puzzling because H1*-H2* is one of the most common acoustic parameters in studies of VQ. We conjecture that the puzzling results may be due to two limitations of the preliminary study: the number of talkers was small and the effect of tones on phonation has not been fully considered.

### Phonation and tones

Recent studies on phonation contrasts demonstrated that non-modal phonation and tone may co-vary in languages (e.g., [19]). Tonal languages differentiate lexical meanings based on the pitch of words. When an individual manipulates the vocal folds to achieve different pitch targets, there are also accompanying changes in phonation. Hence, pitch and phonation types are likely to interact in tonal languages. Phonation contrasts are employed on top of pitch contrasts to distinguish tonal categories in South East Asian tonal languages, such as Vietnamese and Burmese [20]. Non-modal phonation is also found in Chinese dialects, such as breathy phonation in Wu dialects [21], and creaky phonation in the low pitch targets of Mandarin [22] and Cantonese [23]; however the distinctive use of phonation is typically not phonemic in Chinese dialects.

In contemporary Cantonese, creaky voice accompanies the extra-low-level tone (T4), low-level tone (T6), and the lowest point of the high rising tone (T2). Creaky voice is non-phonemic in Cantonese although it was claimed to be found more frequently among male speakers [24]. Yu and Lam [23] examined the occurrences of creaky voice in 12 Cantonese talkers. They found that the creaky voice is most frequent in T4, followed by T6. They further conducted a perception test and found that creaky voice enhanced the identification rate of T4 when pitted against T6. However, a subsequent study by Zhang and Kirby [25] did not find such perceptual effect when the f0 of stimuli is controlled. Hence, the role of creaky phonation on tone identification or perception in Cantonese is not yet conclusive.

### Research goals

Based on the findings of previous studies, this paper explores whether the differences in Cantonese accents may be due to VQ associated with tone. Since VQ characteristics are very individualized, a relatively large sample size is required to obtain statistically meaningful results. Considering the complicated historical relationships between the two cities as described above and the findings in Fung [18], the VQ features of the two accents are quite likely to have changed over time, and the changes may have been reflected in different age groups according to the Apparent Time Framework. This motivates us to examine with more precision the VQ differences between HKC and GZC using a larger sample size of three distinct age groups. The following research questions are examined in this paper: (1) Which acoustic parameter of VQ is best to distinguish the two accents? (2) What are the overall VQ differences between HKC and GZC? (3) How does tonal category associate with the VQ differences between HKC and GZC? (4) What are the changes in VQ over time in Cantonese? (5) What are the differences in frequency and acoustic features of the creaky voice between the two accents?

## Methods

### Ethics statement

The experiments were approved by the Human Subjects Ethics Sub-committee of The Hong Kong Polytechnic University, and conducted according to the Declaration of Helsinki. Informed written consents were obtained from the participants in accordance with the study protocols.

### Talkers

A total of 191 talkers were included in the current study. They were the 120 HKC talkers recruited for the HKC tone merger study [26] and the 71 GZC talkers recruited for the GZC tone merger study [27] conducted in 2009–2012. The talkers were divided into three age groups: young (18–25 years of age), middle-aged (30–45 years of age) and senior (50–65 years of age). All HKC talkers were born and raised in Hong Kong with HKC as their native language and major communication means at home. All GZC talkers were all raised and resided in the four “old districts” of Guangzhou city (i.e. Dongshan, Yuexiu, Haizhu and Liwan) with GZC as their native language and major communication means at home. Only GZC talkers from those four “old” districts were recruited because the geographical boundary of Guangzhou city has changed in the past decades and the population of the “new” districts consists of people originated from other cities who were very likely to speak another Cantonese dialect or accent. Table 2 summarizes the demographic information of the talkers according to their gender and age groups, including the age for each group in means and standard deviations.

**Table 2:** Means and standard deviations of age in years, and number of talkers for each age and gender group of HKC and GZC.

		HKC		GZC	
Age Group	Gender	Mean (SD)	n =	Mean (SD)	n =
**Senior**	**Male**	52.73 (2.45)	11	58.40 (5.03)	5
	**Female**	52.78 (2.33)	9	56.10 (2.47)	10
	**All gender**	52.75 (2.34)	20	56.87 (3.52)	15
**Middle-aged**	**Male**	38.96 (3.36)	25	39.08 (2.78)	12
	**Female**	40.44 (3.52)	25	37.29 (5.27)	14
	**All gender**	39.70 (3.49)	50	38.12 (4.32)	30
**Young**	**Male**	21.60 (1.55)	25	23.71 (3.63)	14
	**Female**	22.04 (1.49)	25	22.50 (1.55)	16
	**All gender**	21.82 (1.52)	50	23.07 (2.74)	30
**All age groups**	**Male**	34.33 (12.04)	61	35.26 (13.01)	31
	**Female**	34.53 (11.86)	59	36.08 (13.80)	40
	**All gender**	34.43 (11.90)	120	35.72 (13.37)	71

### Speech materials

The speech materials were the sentences collected in the read aloud tasks in the previous tone merger studies by Fung and Lee [26] and Ou [27]. The sentences contain 18 target syllables derived by three CV roots /fu/, /ji/ and /si/ in six lexical tones. All derivations were real Cantonese syllables and each of them was embedded in two carrier sentences /ŋɔ13 ji21 ka55 tuk2 X tsi22/ ‘I am now reading the X character’ and /ni55 kɔ33 tsi22 hɐi22 X/ ‘This character is X’. In the first sentence, the target syllable was embedded in a non-final position whereas in the second sentence, the target syllable was in a final position. The complete set of Chinese syllables is provided in the supporting information section.

Although all the target syllables are real Cantonese words, a handful of speakers were unable to produce the target tones due to the on-going tone merging phenomenon in Cantonese [26, 27]. Therefore, prior to data analysis, productions that were judged as failing to meet the target tone in the previous tone merger studies were removed from the current data set. The criteria for judgement of tone mergers were auditorily-based: two research assistants trained in phonetics listened to each production and judged whether it met the target tone. Eventually, a total of 6,203 syllables were included in data analysis of the current study.

### Data analysis

**Acoustic measurement.** The speech materials were manually labelled in Praat [28]. The vowel portions of the target syllables were annotated by the second author based on clear display of the first two formants. The voice quality settings of HKC and GZC were gauged by the following commonly used acoustic parameters, including spectral measures and noise measures, using VoiceSauce v.1.3.1 [29]:

- H1*-H2* (the amplitude differences between the first and second harmonics, and asterisks denote the correction of spectral magnitude estimation influenced by formants frequencies and bandwidths [30])
- H2*-H4* (the amplitude differences between the second and fourth harmonics)
- H1*-A1* (the amplitude differences between the first harmonic and the first formant)
- H1*-A2* (the amplitude differences between the first harmonic and the second formant)
- H1*-A3* (the amplitude differences between the first harmonic and the third formant)
- H4*-H2k* (the amplitude difference between the fourth harmonic and the harmonic near 2kHz)
- H2k*-H5k* (the amplitude difference between the two harmonics near 2kHz and 5kHz)
- Cepstral Peak Prominence (CPP; the difference between the height of the peak relative to the regression line across the overall cepstrum [31])
- Harmonic-to-noise ratios (HNR) at four frequency ranges: 0-500Hz (HNR05), 0-1500Hz (HNR15), 0-2500Hz (HNR25) and 0-3500Hz (HNR35)

Broadly speaking, these acoustic measures have been used to quantify VQ in various languages, although the relationships between the articulatory mechanisms and the acoustic measures are not always clear. In general, spectral measures have been used to quantify glottal tension. For example, H1*-H2* successfully distinguish creaky and modal voice in Mandarin [22], as well as breathy and modal voice in Hmong [32]. This measure was also found to reflect vocal fold stiffness [33] and the degree of glottal opening [34]. H2*-H4* was found to be an acoustic cue for breathiness in Hmong [35]. H1*-A1* was suggested to reflect the degree of posterior glottal opening [36], whereas H1*-A2* and H1*-A3* both reflected the abruptness of glottal closure [36, 37]. H4*-H2k* and H2k*-H5k* were used to examine phrase-final creak in English [38]. On the other hand, the noise measures were broadly associated with breathiness or airflow at the glottis. For example, CPP was proposed as a measure of the periodicity of acoustic signal [31]. In that same study, CPP was found to distinguish modal and breathy phonation, a non-modal phonation characterized by increased noise in perception. Nevertheless, noise may also arise from creaky voice which display aperiodic acoustic signals [16]. The other noise measures, Harmonic-to-noise ratio (HNR), measure the noise components of the harmonics at four frequency ranges from 0 to 3500Hz in the spectrum [39]. The various HNR measures were found to distinguish the modal voice versus the breathy voice of high and low registers in Shanghainese [21].

In addition, we obtained f0 values measured from the Straight algorithm in VoiceSauce. This allows us to examine whether f0 height contributes to the distinction of HKC and GZC in our subsequent analysis. The acoustic measurements were time-normalized into 11 equal segments in VoiceSauce, and then scaled within individual speaker’s values from -1 to 1. Results of the first three and the last three segments were removed to minimize any carry-over or sentence-final effects (i.e. the middle five segments were used for subsequent analysis). A mean value was then computed for each acoustic parameter by averaging the values in each token. The mean values were used for statistical analysis.

#### Creaky voice annotation

Manual annotation of creaky voice was conducted by the second author on the 6,203 syllables. A binary decision on the presence of creaky voice was made based on the auditory criterion adopted by Yu and Lam [23], and the displays of the speech waveform, both wideband and narrowband spectrograms, as well as F0 contours. Specifically, the production was annotated as creaky if it exhibited the auditory quality of creakiness, and the visual display of at least one of the following features: (i) consecutive missing f0 values based on the default f0 tracking algorithm in Praat; (ii) strong damping, irregular or alternating cycles of glottal pulses; (iii) strong subharmonic structures in the narrowband spectrogram. Since some of the target syllables were produced at the sentence-final position, which may exhibit sentence-final creak, only productions showing creakiness over one-third of the syllable were considered as creaky.

### Statistical analysis

To evaluate the relative contribution of individual acoustic parameter to the overall phonetic distinction between HKC and GZC, a linear discriminant analysis was performed using the MASS package in R [40, 41]. Pearson-r correlation was then applied to measure the correlation between the linear discriminant and the various acoustic parameters. Similar methods have been used for evaluating the contribution of acoustic parameters to phonation contrasts in Yi [42], Shanghainese [21] and Mazatec [43]. This procedure was expected to yield the most useful acoustic parameter that distinguishes the voice quality between HKC and GZC.

We then fitted a linear mixed effect model for the acoustic parameter that was found best to distinguish voice quality of HKC from GZC, using the lme4 package [44]. Details of model construction are presented in the result section below. Post-hoc analysis was performed through least-square means analysis with the lsmeans package [45], which allowed pairwise comparisons of the various fixed effects that we are interested in, such as age group and accent. *P*-values in the post-hoc analysis were adjusted using the Tukey method.

For analysis of creaky voice, we fitted a binomial mixed effect model to determine whether the frequency of creaky voice differ between tones, accents and age groups. We also fitted a linear mixed effect model to examine the differences in the acoustic characteristics of creaky voice in HKC and GZC. Details of model construction are described in the result section below.

## Results

### Voice quality differences between HKC and GZC

**Fig 2:**
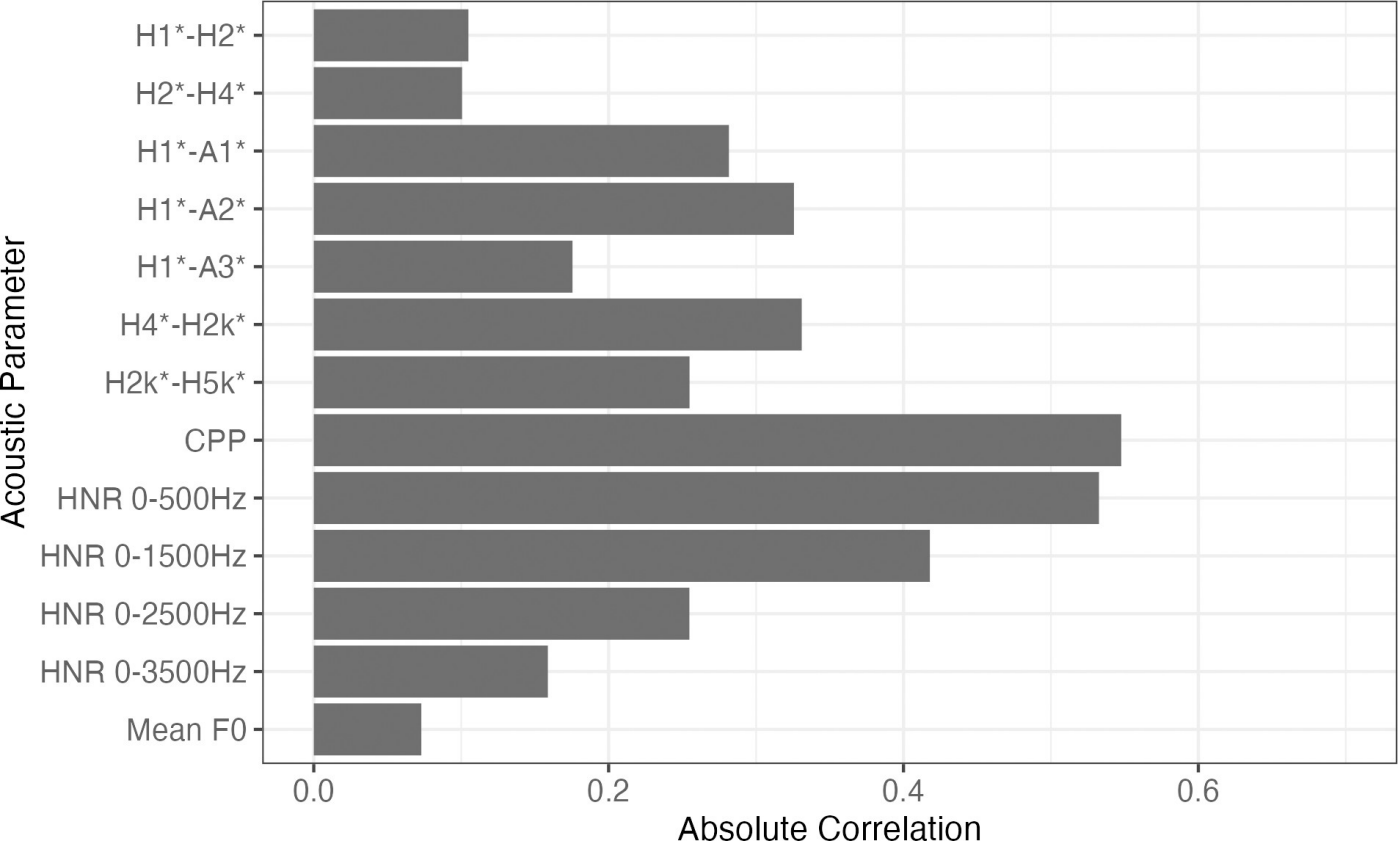
Absolute correlations of the acoustic parameters and the linear discriminant. This shows the relative contribution of the acoustic parameters in distinguishing HKC and GZC.

Our first research question asks which acoustic parameter best contributes to the distinction in VQ between HKC and GZC. We conducted a linear discriminant analysis, and measured the correlation between the linear discriminant and the acoustic parameters used in this study. Fig 2 shows the absolute correlation between the linear discriminant and the acoustic parameters. It is apparent that CPP contributed the most to the distinction of HKC and GZC, as it showed the highest correlation with the linear discriminant (*r* = 0.55). The other noise measures, HNR05 and HNR15, also showed substantial contribution (*r* = 0.54 and 0.41, respectively) to the distinction of HKC and GZC. As for the spectral measures, H1*-A1* and H1*-A2* showed the highest correlations with the discriminant value (*r* = 0.33 and 0.36, respectively). On the other hand, H1*-H2* showed a relatively low contribution in distinguishing the two Cantonese accents, although it is arguably the most commonly used acoustic parameter in the studies of linguistic VQ. Furthermore, mean f0 had the lowest correlation to the linear discriminant, suggesting that f0 height did not contribute to the distinction of HKC and GZC. Taken together, the main differences in VQ between HKC and GZC lie in the noise component (as reflected by CPP and HNR measures). As CPP shows the most contribution to distinguishing HKC and GZC, we will focus on the results of CPP in the following sections.

Our second research question asks what are the overall VQ differences between HKC and GZC? Since CPP was the best detector of the acoustic distinction of HKC and GZC, we fitted a linear mixed effect model on CPP to evaluate the effects of accent, gender, age groups and lexical tones. The initial model included the random intercepts of TALKER, SYLLABLE TYPE (/fu/, /ji/ or /si/) and SYLLABLE POSITION (non-final or sentence final). The fixed effects of TONE, GENDER, ACCENT and AGE (i.e., age group) were added to the model in a stepwise manner. We then added the interaction of TONE*ACCENT, as well as TONE*ACCENT*AGE. As gender variation in voice quality is not the focus of the current study, we did not include the interaction of GENDER with the other fixed effects in the model. Best model fit were established based on log-likelihood tests compared to the next-simplest model. Estimates of the fixed effects were measured through ANOVA.

The best fitted model included the main effects of TONE (*F*[5,6015] = 419.16, *p* = 0.001), GENDER (*F*[1,190] = 12.56, *p* <0.001), ACCENT (*F*[1,191] = 26.2, *p* <0.001) and AGE (*F* [2,191] = 2.86, *p* <0.06), as well as the interaction of TONE*ACCENT (*F*[5,6015] = 3.46, *p* = 0.004), and TONE*ACCENT*AGE (*F*[10,6015] = 2.32, *p* = 0.01). Results of the full model are included in the supporting information section. The results suggest that there was an overall accent difference in VQ between HKC and GZC, when age, gender and lexical tones are controlled for. Specifically, HKC had a higher CPP than GZC (HKC: *M* = 0.125, *SD* = 0.379; GZC: *M* = 0.026, *SD* = 0.391), which suggests that HKC had less noise than GZC. The model also showed significant interaction effects of TONE*ACCENT and TONE*ACCENT*AGE. This suggests that the VQ of HKC and GZC varied across different tones and age groups. Details of the interaction of tone category and accent should be in order.

**Fig 3:**
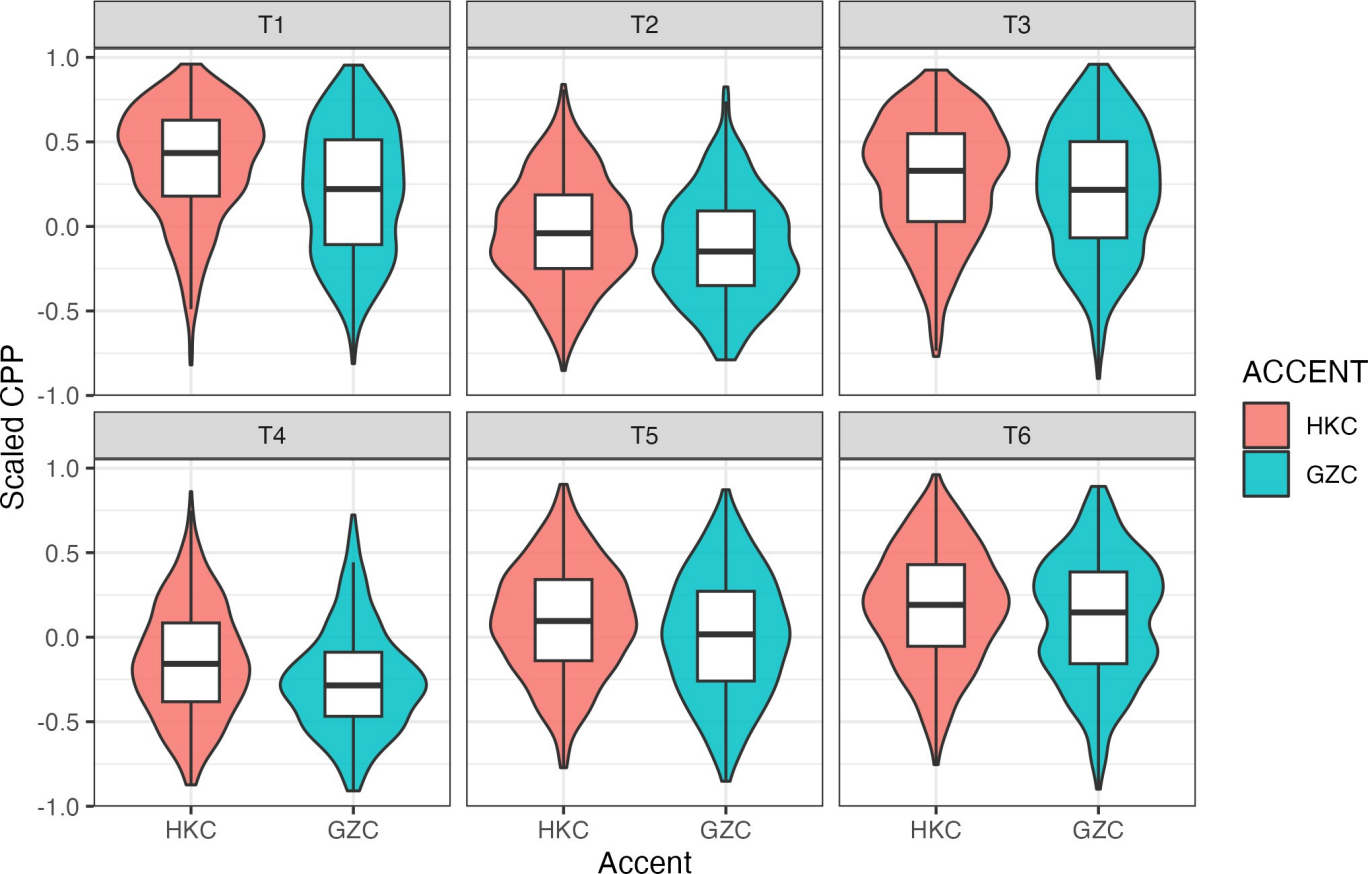
Violin and boxplots of CPP across the six lexical tones in HKC and GZC.

Our third research question aims to examine if the VQ differences in HKC and GZC are associated with tonal categories. Fig 3 shows the violin plots of CPP across the six lexical tones in HKC and GZC, together with boxplots showing the means and standard deviations. The post-hoc pairwise comparisons showed that VQ of all six tones were significantly different between the two accents. The full results of the post hoc analysis are provided in the supporting information. Overall, HKC had higher CPP values than GZC for all six tones, which suggest that HKC had decreased noise than GZC across all tones. Furthermore, the magnitude of VQ differences varied across the six tones: T3 had the smallest difference between the two accents (β = -0.072, *t* = -2.373, *p* = 0.018), followed by T6 (β = -0.109, *t* = -3.493, *p* = 0.001). T2 and T5 showed similar magnitude of differences in CPP between HKC and GZC (T2: β = -0.127, *t* = -4.291, *p*<0.001; T5: β = -0.135, *t* = -4.315, *p* <0.001), followed by T4 (β = -0.147, *t* = -4.878, *p* <0.001). T1 showed the largest difference in CPP between HKC and GZC (β = -0.173, *t* = -5.835, *p* <0.001). In sum, the largest differences in CPP between the two Cantonese accents were found in the highest and the lowest level tones (T1 and T4), followed by the two rising tones (T2 and T5), and finally, the mid- and the low- level tones (T3 and T6).

### Voice quality features among age groups

**Fig 4:**
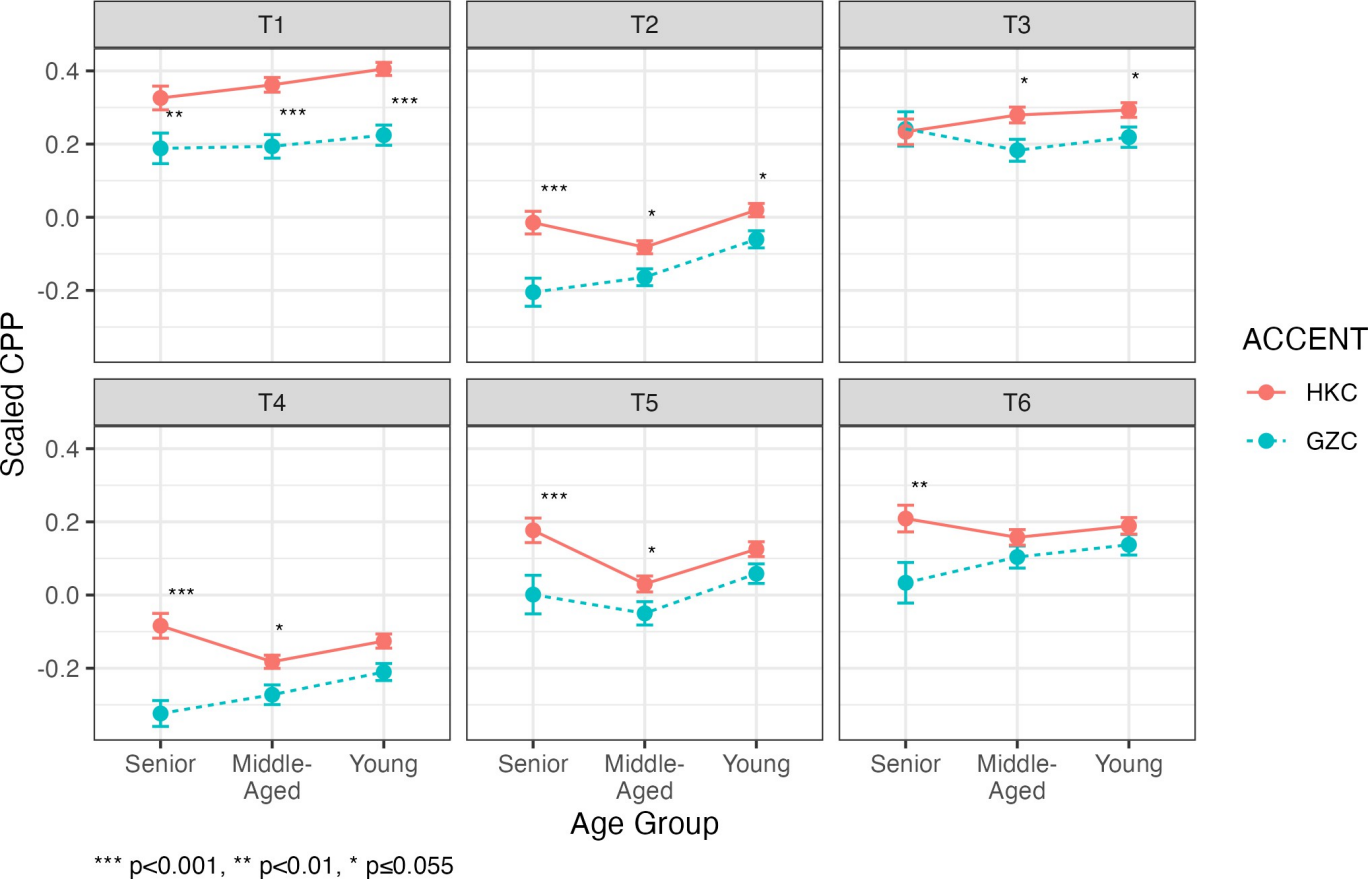
Mean CPP across tones, accents, and age groups. HKC is represented by pink circles and solid line, whereas GZC is represented by light blue circles and dashed line. The error bars represent the standard errors of the means. Asterisks denotes significant differences found between the two accents within that age group, based on the post-hoc pairwise comparisons.

The regression model also provided answer to our fourth question which asks: what are the changes in VQ over time in Cantonese? The significant interaction effect of TONE*ACCENT*AGE (*F*[10, 6015] = 2.3, *p* = 0.01) on CPP suggests that the voice quality differences between HKC and GZC were mediated by age groups. The mean CPP of the six tones across age groups of HKC and GZC are illustrated in Fig 4.

#### T1 (high-level tone)

Comparing the two accents in each age group, all three age groups maintained the accent differences in CPP (Senior: β = -0.166, *t* = -2.61, *p* = 0.009; Middle-aged: β = -0.169, *t* = -3.743, p <0.001; Young: β = -0.184, t = -4.317, *p* <0.001). There was a slight increase in the differences of CPP from senior group to the young group suggesting that the distinction in T1 voice quality increased over time.

#### T2 (high-rising tone)

The overall trend showed that the voice quality difference in T2 between HKC and GZC reduced over time. T2 had a larger magnitude of difference in the senior group (β = -0.21, *t* = -3.3, *p* = 0.001), compared to the middle-aged (β = -0.086, *t* = -1.921, *p* = 0.055) and the young (β = -0.085, *t* = -1.996, *p* = 0.047). Although the p-value in the middle-aged group slightly exceeded the alpha level of 0.05, due to the exploratory nature of this study, we take this case as a positive evidence of differences between the two Cantonese accent. In any cases, the two young groups maintained the accent difference in T2.

#### T3 (mid-level tone)

There was no accent difference in T3 in the senior group (β = -0.028, *t* = -0.423, *p* = 0.672). However, there was accent differences in the middle-aged (β = -0.101, *t* = -2.247, *p* = 0.025) and in the young (β = -0.086, *t* = -1.995, *p* = 0.047). This suggests that the accent difference in T3 became prominent in the middle-age and was maintained by the young.

#### T4 (extra-low-level tone)

For T4, the difference in CPP between HKC and GZC gradually diminished across age groups. The largest difference was found in the senior group (β = -0.264, *t* = -4.08, *p* <0.001), followed by the middle-aged (β = -0.97, *t* = -2.113, *p* = 0.035). The differences in the young group was not significant (β = -0.08, *t* = -1.851, *p* = 0.065), suggesting that the VQ of T4 in HKC and GZC merged in the young.

#### T5 (low-rising tone)

T5 showed a similar pattern to T4: the accent difference diminished across age groups. The senior groups showed the largest difference between HKC and GZC (β = -0.222, *t* = -3.295, *p* = 0.001), followed by the middle-aged (β = -0.108, *t* = -2.275, *p* = 0.023). There was no significant accent difference in T5 in the young (β = -0.073, *t* = -1.673, *p* = 0.095).

#### T6 (low-level tone)

As for T6, only the senior group showed a difference in CPP between accents (β = -0.189, *t* = -2.766, *p* = 0.006). Both the middle-aged and the young groups did not show significant accent differences in T6 (Middle-aged: β = -0.064, *t* = -1.371, *p* = 0.171; Young: β = -0.076, *t* = -1.703, *p* = 0.089).

Let us summarize the differences between age groups from the perspective of the changes in voice quality features under the Apparent Time Framework, which assumes the senior generation represents the earlier form of the language and the younger generations represent a later form. Under this perspective, three patterns were observed:

1. Distinctive VQ was maintained, as attested by the two high tones: T1 and T2. The voice quality differences between HKC and GZC is maintained in each age group for the two high tones.
2. A difference in voice quality was developed, as attested by mid-level tone (T3). There was no significant difference between the two accents in the senior, but the middle-aged and the young became dissimilar.
3. Distinctiveness in voice quality diminished across age groups, as attested by the low tones: T4, T5 and T6. For these three tones, there were accent differences in the senior and the middle-aged, but the young became not different from each other.

### Creaky voice in HKC and GZC

#### Prevalence of creaky voice

**Fig 5:**
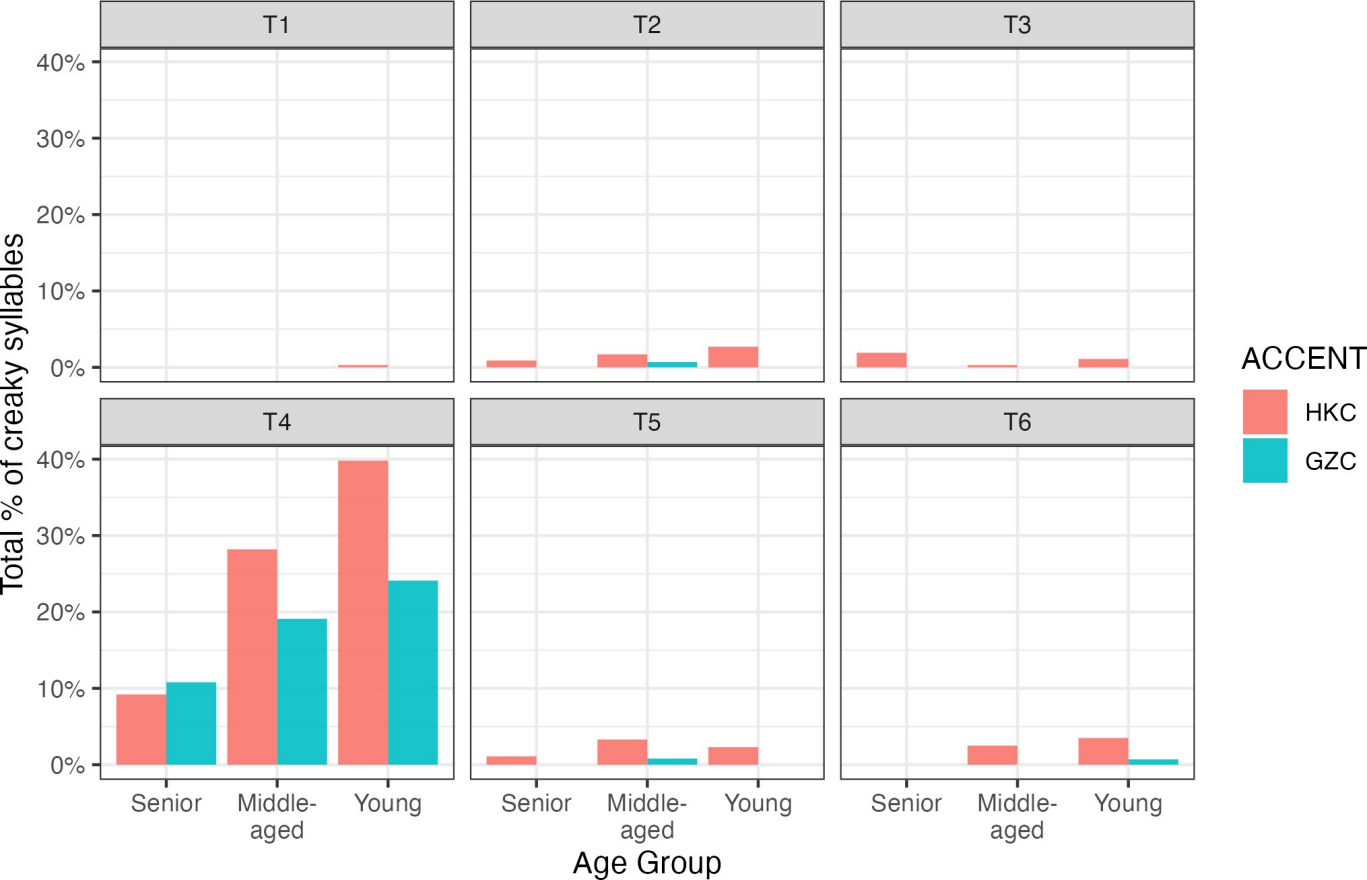
The percentage of creaky syllables across tones, age groups and accents.

Our fifth research questions ask: What are the differences in frequency and acoustic feature of the creaky voice between the two accents? From the total of 6,203 syllables analyzed, 335 of them were found creaky (= 5.4%). The percentage of creaky syllables across the six tones are shown in Fig 5. We fitted a binomial mixed effect model to determine if the log-odds of creaky voice occurrences differ across tones, accents, gender and age groups. The initial model included a random intercept of TALKER, SYLLABLE TYPE and SYLLABLE POSITION. The response variable is the occurrence of creaky voice coded as 1 (= creaky) and 0 (= non-creaky). We then added the fixed effects of TONE, GENDER, ACCENT and AGE, as well as the interaction effects TONE*ACCENT and TONE*ACCENT*AGE in a stepwise manner. Significance of model was established based on better fit than the next simplest-model. We set T4 as the reference level for TONE because creaky voice is mostly associated with this low-level tone [23].We also set the middle-aged as the reference level for AGE, which should facilitate the comparison of seniors with the middle-aged, and the young with the middle-aged. Full results of the best-fitted model is provided in the supporting information section. The best fitted model included the main effects of TONE, ACCENT and AGE. There was no main effect of GENDER and no interaction effect. For the effect of tone, all other tones were significantly different from T4 in the log-odds of presence of creaky voice (all *p* <0.001). This suggests that creaky voice is mainly tied in to T4 in Cantonese. For the effect of accent, it was found that HKC had higher occurrences of creaky voice than GZC (*β* = 1.013, *z* = 3.12, *p* = 0.002). Finally, for the effects of age groups, the seniors had lower log-odds of presence of creaky voice (*β* = -1.441, *z* = -3, *p* = 0.003) than the middle-aged, whereas the young were not different from the middle-aged (β = 0.494, *z* = 1.53, *p* = 0.127). This suggests that the middle-aged and the young exhibit similar log-odds in the presence of creaky voice in Cantonese. Our next step was to examine whether there are any differences in the acoustic features of creaky voice between HKC and GZC.

#### Acoustic features of creaky voice

In addition to the differences in the frequency of creaky voice, we also wondered if HKC and GZC exhibit differences in the acoustic features of creaky voice. Hence, we extracted the creaky syllables of T4 and fitted a mixed effect model to examine if there was an effect of accent, gender and age groups on CPP. The model also included the random effects of talker, syllable type and syllable position. The best fitted model included an ACCENT effect on CPP (β = 0.107, *t* = 2.46, *p* = 0.016) but not GENDER and AGE effect. Similar to the trend of overall voice quality in T4, creaky syllables in HKC showed higher CPP than GZC, suggesting that GZC talkers produced creaky voice that had increased noise than HKC. However, as mentioned in [16], certain subtypes of creaky voice may also involves increased noise, such as aperiodic voice and non-constricted creak. Therefore, to examine whether the low CPP of creaky voice in GZC was induced by breathiness, we also fitted a linear mixed effect model on H1*-H2*, which is the canonical measure of creaky voice and glottal tension in the literature. The construction of this model was essentially the same as for CPP, except that the response variable is H1*-H2*. The best fitted model showed an effect of ACCENT (β = 0.12, *t* = 2.38, *p* = 0.02) and GENDER (β = -0.29, *t* = -6.33, *p* <0.001), but no effect of age. Interestingly, the results of H1*-H2* showed a different direction compared to CPP: GZC had a lower H1*-H2* than HKC (GZC: *M* = -0.234, *SD* = 0.33; HKC: *M* = -0.104, *SD* = 0.33), which suggests that GZC was tenser than HKC in their production of creaky syllables. Taken together, the low CPP of creaky syllables in GZC was likely caused by increased glottal tension, rather than breathiness.

**Fig 6:**
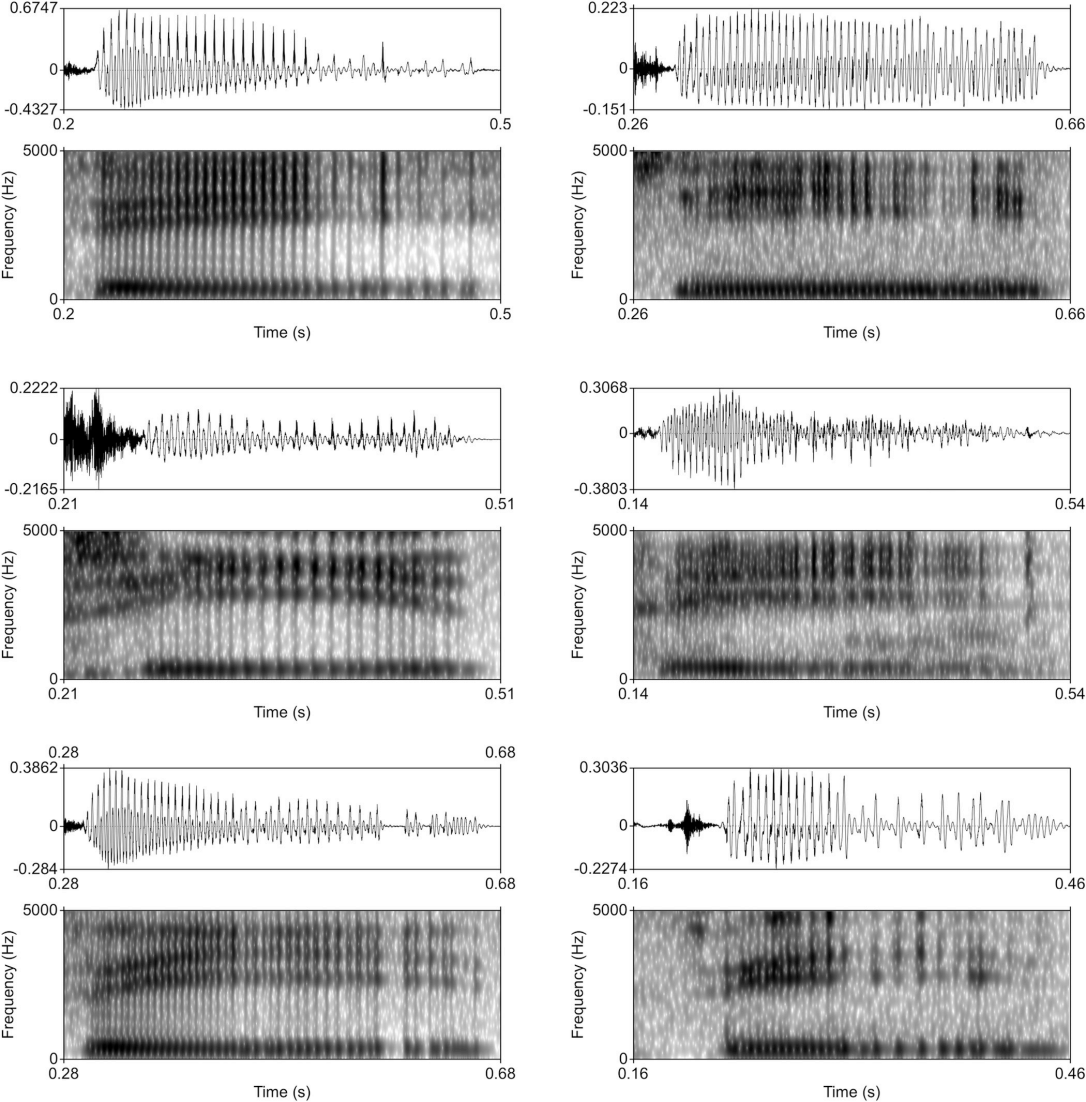
Waveform and spectrogram of creaky voice of /si21/ in a senior female talker of HKC (top left panel) and of GZC (top right panel), a middle-aged female talker of HKC (middle left panel) and of GZC (middle right panel), and a young female talker of HKC.

Moreover, the acoustic differences of creaky voice between the two accents were supported by waveform and spectrographic displays of tokens of creaky T4. Fig 6 shows the waveforms and spectrograms of six selected examples of creaky T4 syllables in HKC and GZC. As revealed from the spectrograms, creaky syllables in GZC creaky syllables were characterized by widely-spaced and irregular vocal pulses, which suggest that the vocal folds were tightly adducted, compared to the creaky voice of HKC.

## Discussion

This study examined the phonetic differences between HKC and GZC in the domain of VQ in the hope of providing answers to the five research questions.

Our first question asks what acoustic parameter of voice quality best distinguish the two Cantonese accents. Results from the linear discriminant analysis demonstrated that CPP contributed the most to the distinction between HKC and GZC. As CPP is a measure of noise component of the voice, this suggests that the main difference between HKC and GZC lied in the degree of glottal noise, rather than glottal tension, which would be quantified by spectral parameters such as H1*-H2*. The LDA results also showed that f0 had negligible contribution to the distinction of HKC and GZC, consistent with the findings in previous studies. In the study by Wu [4], there was no apparent f0 differences found in the six tones of HKC and GZC. In Fung’s study [18], it was found that the HKC has wider pitch range than GZC, but no difference in pitch level. Our results provided further evidence that the difference between the two Cantonese accents is not attributed to tonal height, but rather the VQ aspect of tonal production.

Our second question asks what are the overall VQ differences between HKC and GZC. Our mixed effect model showed that GZC had lower CPP than HKC, which indicated increased noise in the overall VQ of GZC. However, the acoustic evidence of CPP alone would not allow us to conclude whether such noise was caused by breathiness or by glottal tension. In Fung’s study [18], which was based on a smaller sample size of the same group of participants, it was found that the results from H2*-H4*, H1*-A1*, H1*-A2*, H1*-A3* and CPP all pointed towards GZC being breathier than HKC, a conclusion based on the breathy-creaky continuum of VQ. Nevertheless, further investigation is necessary to determine the source of noise in GZC, and the perceived voice quality in the two Cantonese accents.

The statistical model also provided answers to our third question, which asks if the VQ differences in HKC and GZC are associated with tonal categories. Our findings demonstrated that lexical tone was a crucial factor mediating the VQ difference of HKC and GZC. Although GZC had overall increased noise than HKC, the extent of CPP difference varied across tones. The largest CPP differences were found in T1 and T4, the highest and the lowest tone in Cantonese. This is followed by T2 and T5 (the two rising tones), whereas T3 and T6 (the mid- and low-level tones) showed the least CPP differences between accents. It implies that the VQ difference between the two accents should be largely contributed by the two extreme tones in Cantonese. As shown in previous studies, non-modal phonation was also attested in some Cantonese tones with low pitch target. Creaky voice was highly tied to T4 in Cantonese. This is true for both HKC and GZC. Similar to Mandarin, creaky voice in Cantonese should be a feature driven by low f0 target. As Kuang [22] remarked: “pitch-driven non-modal phonation tends to occur when pitch production exceeds certain limits, as substantial changes in the glottal configurations and vibratory patterns have to be made for the highest and lowest pitch targets.” In Cantonese, such drastic changes in glottal configuration led to creaky phonation in the tonal category with the lowest pitch target.

Our fourth question asks if there are any changes in VQ over time in Cantonese. Our study revealed that VQ varied across age groups in HKC and GZC. This was further complicated by the relationship between VQ and lexical tones. Specifically, we found that the VQ of the young talkers of the two accents were merging in the low tones: T4, T5 and T6, while the differences in VQ was maintained across age groups in the non-low tones: T1, T2 and T3. Under the Apparent Time Framework, our findings were consistent with the historical development of the two accents portrayed in the introduction section. The speech output of the senior talkers and the middle-aged talkers may reflect the speech form in the *separated* stage when the contact of the two cities were cut. The two accents developed their own VQ features in this stage. The speech output of the young talkers may reflect the speech form in the *reunified* stage when the two cities were reconnected. GZC might have been highly influenced by HKC in recent years through the propagation of Hong Kong pop culture. As for HKC, the influence of GZC might be due to population mobility. As a city of immigrants, Hong Kong has received large number of immigrants originated in Mainland China, most of whom have lived or worked in the urbanized cities of the Pearl River Delta regions and could speak some degree of GZC. When they migrated to Hong Kong, there might have been a levelling of HKC and GZC accents. This is to say, the “reunification” of the two accents revealed among the young Cantonese talkers may be caused by two different routes: In GZC, the accent levelling may be caused by the indirect contact of HKC through culture propagation. In HKC, the accent levelling may be caused by direct contact of GZC through population movements. As to why Cantonese tones did not exhibit the same trend of change in voice quality, this was likely due to the development of the Cantonese tonal system. As reviewed in the introduction section, T4, T5 and T6 were developed from T1, T2 and T3 respectively when Cantonese went through devoicing in the initial consonants. In other words, the three low tones were the most recently evolved in the history of Cantonese. The merging of the VQ of two accents may have started from these relatively “new” tonal categories. The implication of this finding should be a very interesting topic to pursue from the perspective of diachronic sound change.

Our last question asks what are the differences in frequency and acoustic features in creaky voice between the two Cantonese accents. We found that HKC had higher frequency of creaky tokens than GZC in T4, suggesting that the prevalence of creaky voice was mediated by accent. In addition, we also found that the creaky voice of the two Cantonese accents may belong to different subtypes since different acoustic features were attested in their creaky tokens. Our acoustic analysis showed that HKC had higher CPP than GZC in the T4 syllables that display creakiness, similar to the overall trend of the VQ of all T4 syllables of the two accents. This indicated increased noise in the creaky voice of GZC. Nevertheless, when we further examined H1*-H2*, a spectral tilt measure, to determine whether the increased noise in creaky T4 of GZC was induced by breathiness, we found that GZC had lower H1*-H2* than HKC. This result suggests that GZC had increased glottal tension than HKC. We opine that the increased noise in creaky voice of GZC is potentially induced by high glottal tension, rather than breathiness. These findings were consistent with the spectrographic display, which suggested that GZC had increased glottal tension than HKC, due to its widely-spaced and irregular glottal pulses. Nonetheless, the physiology of creaky voice is not very clear. For instance, Laver [46] described creaky voice as involving high level of adductive laryngeal tension (i.e. strong muscular tension to bring the vocal folds together). However, Esling and Edmondson [13, 47] stated that creaky voice was produced with very relaxed vocal folds, allowing a loose enough glottis that vibration is slow and undulating. Further investigations on the relationship of articulatory mechanisms and acoustic measures are necessary for a better understanding of the subtypes of creaky voice in Cantonese.

Finally, our analysis also showed that creaky voice changed over time. The frequency of creaky voice was increasing from senior to young talkers in both accents. As illustrated in Fig 5, the percentage of creaky voice in T4 increased drastically in HKC young from the middle-aged (28% to 40%), whereas in GZC such increase occurred to a lower extent (19% to 24%). Although there was no interaction between accent and age in the statistical model, it is possible that the prevalence of creaky voice increases with a faster rate in HKC. The prevalence of creaky voice in the two accents also showed a different trend from that of the overall voice quality features of all T4 tokens, in which we found the two accents showed no CPP difference in the young. This may be due to the large variability of creaky voice occurrences among individuals, which was also attested in Yu and Lam’s study [23] with talkers of roughly the same age group. We suspect that the widespread of creaky T4 syllables of HKC may be influenced by the popularity of creaky voice in American English (e.g. [48, 49]). This is supported by the sociophonetic study on the influence of English on HKC voice quality reported in Fung and Lee [17]. The authors found that higher-educated female HKC speakers displayed a creaky voice quality, which may be attributed to the influence of American English promoted by pop culture. As suggested by one of the anonymous reviewers of this paper, another possible explanation of the widespread of creaky voice in HKC is that creaky voice is becoming a primary cue to the perception of T4 in HKC, where it occurred more frequently. As argued by Yu and Lam [23], creaky voice may be an acoustic cue to facilitate the identification of T4.

## Conclusion

In conclusion, this study explored the phonetic differences between two accents of Cantonese from the domain of VQ. We have found that HKC had a higher CPP than GZC, suggesting that the overall voice quality of GZC exhibit increased noise compare to HKC. We have also demonstrated that VQ varied in the realization of different tonal targets. The two accents showed the greatest VQ differences in the two extreme tones: the high-level tone (T1) and extra-low-level tone (T4). Furthermore, creaky voice was mainly tied to T4 in Cantonese, and HKC had a higher frequency of creaky voice than GZC. In addition to frequency, the acoustic feature of creaky voice were also different between the two accents. Creaky voice in GZC was characterized by increased noise and increased tension, which suggest that HKC and GZC are likely to exhibit different subtypes of creaky voice. Last but not least, our study has also revealed that age was a mediating factor in the voice quality of the two accents. In particular, the VQ of the young generations of the two accents have started merging from the three low tones. The prevalence of creaky voice was increasing over time in both accents. To the best of our knowledge, this is the first study to document accent differences and evolution in Chinese languages from the perspective of VQ. The rich acoustic data of the VQ of different tonal categories and different age groups presented in this study will facilitate and promote future perceptual investigations on which phonetic cues do native speakers use to discern the two accents. While our study has made significant strides in understanding VQ in Cantonese based on sentence reading, our future research will further explore the VQ of continuous speech in the two accents. In short, this study does not only fill the gap in sociophonetics and Chinese dialectology, it will open up a range of explorations for phoneticians, speech pathologists and speech engineers.

## Supporting information

S1 File Appendix. (DOCX)

S2 File Data. (CSV)
