# Supplementary material for: Separated and reunified: An apparent time investigation of the voice quality differences between Hong Kong Cantonese and Guangzhou Cantonese
Source: PLoS One. 2023 Oct 18;18(10):e0293058. doi: 10.1371/journal.pone.0293058 (PMC10584129; doi:10.1371/journal.pone.0293058)
Supplement: S1 File — (DOCX) [file pone.0293058.s001.docx]

**S1 Appendix**

1. Word list

|  | HKC | | | GZC | | |
| --- | --- | --- | --- | --- | --- | --- |
| Tone | /fu/ | /ji/ | /si/ | /fu/ | /ji/ | /si/ |
| T1  [55/53] | 呼  ‘to call’ | 醫  ‘to cure’ | 師  ‘teacher’ | 夫  ‘husband’ | 衣  ‘clothes’ | 詩  ‘poem’ |
| T2  [35] | 苦  ‘bitter’ | 椅  ‘chair’ | 史  ‘history’ | 苦  ‘bitter’ | 椅  ‘chair’ | 史  ‘history’ |
| T3  [33] | 富  ‘rich’ | 意  ‘intention’ | 嗜  ‘hobby’ | 富  ‘rich’ | 意  ‘intention’ | 嗜  ‘hobby’ |
| T4  [21/11] | 扶  ‘hold’ | 兒  ‘son’ | 時  ‘time’ | 扶  ‘hold’ | 移  ‘move’ | 時  ‘time’ |
| T5  [23] | 婦  ‘woman’ | 耳  ‘ear’ | 市  ‘city’ | 婦  ‘woman’ | 耳  ‘ear’ | 市  ‘city’ |
| T6  [22] | 付  ‘to pay’ | 二  ‘two’ | 示  ‘notice’ | 父  ‘father’ | 二  ‘two’ | 事  ‘event’ |

2. Correlation of linear discriminant and acoustic parameters

| H1*-H2* | 0.1049 |
| --- | --- |
| H2*-H4* | -0.1006 |
| H1*-A1* | -0.2816 |
| H1*-A2* | -0.3257 |
| H1*-A3* | -0.1755 |
| H4*-H2k* | 0.331 |
| H2k*-H5k* | -0.2549 |
| CPP | 0.5477 |
| HNR05 | 0.5326 |
| HNR15 | 0.4179 |
| HNR25 | 0.2548 |
| HNR35 | 0.1588 |
| Mean F0 | -0.07295 |

3. ANOVA results of linear mixed model predicting CPP

Formula: CPP ~ Tone+ GENDER+ ACCENT+ AGE+ TONE*ACCENT+ TONE*AGE+ ACCENT*AGE+ TONE*ACCENT*AGE+ (1|TALKER)+ (1|SYLLABLE)+ (1|POSITION)

|  | Sum Sq | Mean Sq | df1 | df2 | *F* | *p* |
| --- | --- | --- | --- | --- | --- | --- |
| TONE | 156.2 | 31.24 | 5 | 6015 | 419.16 | <.001 |
| GENDER | 0.9 | 0.94 | 1 | 190 | 12.56 | <.001 |
| ACCENT | 2.0 | 1.95 | 1 | 191 | 26.20 | <.001 |
| AGE | 0.4 | 0.21 | 2 | 191 | 2.86 | 0.060 |
| TONE:ACCENT | 1.3 | 0.26 | 5 | 6015 | 3.46 | 0.004 |
| TONE:AGE | 1.5 | 0.15 | 10 | 6015 | 2.01 | 0.029 |
| ACCENT:AGE | 0.1 | 0.07 | 2 | 191 | 0.90 | 0.409 |
| TONE:ACCENT:AGE | 1.7 | 0.17 | 10 | 6015 | 2.32 | 0.010 |

4. Post-hoc analysis for tone*accent, Tukey adjusted p-values

|  | Estimate | SE | df | *t* | *p* |
| --- | --- | --- | --- | --- | --- |
| T1 | -0.173 | 0.0296 | 387 | -5.835 | <.001 |
| T2 | -0.127 | 0.0297 | 389 | -4.291 | <.001 |
| T3 | -0.072 | 0.0301 | 412 | -2.373 | 0.018 |
| T4 | -0.147 | 0.0301 | 411 | -4.878 | <.001 |
| T5 | -0.135 | 0.0312 | 470 | -4.315 | <.001 |
| T6 | -0.109 | 0.0312 | 473 | -3.493 | 0.001 |

5. Post-hoc analysis for accent | tone*age, Tukey adjusted p-values

| Age group | Tone | Estimate | SE | df | *t* | *p* |
| --- | --- | --- | --- | --- | --- | --- |
| Senior | T1 | -0.166 | 0.064 | 385 | -2.610 | 0.009 |
|  | T2 | -0.210 | 0.064 | 391 | -3.301 | 0.001 |
|  | T3 | -0.028 | 0.065 | 433 | -0.423 | 0.672 |
|  | T4 | -0.264 | 0.065 | 415 | -4.080 | <0.001 |
|  | T5 | -0.222 | 0.067 | 485 | -3.295 | 0.001 |
|  | T6 | -0.187 | 0.068 | 491 | -2.766 | 0.006 |
| Middle-aged | T1 | -0.169 | 0.045 | 396 | -3.743 | <0.001 |
|  | T2 | -0.086 | 0.045 | 388 | -1.921 | 0.055 |
|  | T3 | -0.101 | 0.045 | 393 | -2.247 | 0.025 |
|  | T4 | -0.097 | 0.046 | 420 | -2.113 | 0.035 |
|  | T5 | -0.108 | 0.048 | 485 | -2.275 | 0.023 |
|  | T6 | -0.064 | 0.047 | 463 | -1.371 | 0.171 |
| Young | T1 | -0.184 | 0.043 | 383 | -4.317 | <.001 |
|  | T2 | -0.085 | 0.043 | 387 | -1.996 | 0.047 |
|  | T3 | -0.086 | 0.043 | 390 | -1.995 | 0.047 |
|  | T4 | -0.080 | 0.043 | 396 | -1.851 | 0.065 |
|  | T5 | -0.073 | 0.044 | 425 | -1.673 | 0.095 |
|  | T6 | -0.076 | 0.045 | 448 | -1.703 | 0.089 |

6. Means and standard deviations of CPP for tone, accent and age groups

| Tone | Accent | Age Group | Mean | SD |
| --- | --- | --- | --- | --- |
| T1 | GZC | Senior | 0.188 | 0.392 |
|  |  | Middle-aged | 0.194 | 0.390 |
|  |  | Young | 0.224 | 0.369 |
|  | HKC | Senior | 0.326 | 0.353 |
|  |  | Middle-aged | 0.362 | 0.342 |
|  |  | Young | 0.405 | 0.309 |
| T2 | GZC | Senior | -0.205 | 0.354 |
|  |  | Middle-aged | -0.164 | 0.283 |
|  |  | Young | -0.060 | 0.310 |
|  | HKC | Senior | -0.015 | 0.336 |
|  |  | Middle-aged | -0.082 | 0.303 |
|  |  | Young | 0.020 | 0.314 |
| T3 | GZC | Senior | 0.241 | 0.403 |
|  |  | Middle-aged | 0.183 | 0.366 |
|  |  | Young | 0.219 | 0.370 |
|  | HKC | Senior | 0.234 | 0.358 |
|  |  | Middle-aged | 0.279 | 0.367 |
|  |  | Young | 0.293 | 0.337 |
| T4 | GZC | Senior | -0.324 | 0.304 |
|  |  | Middle-aged | -0.272 | 0.307 |
|  |  | Young | -0.210 | 0.303 |
|  | HKC | Senior | -0.084 | 0.371 |
|  |  | Middle-aged | -0.182 | 0.309 |
|  |  | Young | -0.126 | 0.329 |
| T5 | GZC | Senior | 0.001 | 0.422 |
|  |  | Middle-aged | -0.050 | 0.348 |
|  |  | Young | 0.058 | 0.334 |
|  | HKC | Senior | 0.177 | 0.323 |
|  |  | Middle-aged | 0.030 | 0.316 |
|  |  | Young | 0.125 | 0.330 |
| T6 | GZC | Senior | 0.034 | 0.437 |
|  |  | Middle-aged | 0.104 | 0.333 |
|  |  | Young | 0.137 | 0.347 |
|  | HKC | Senior | 0.209 | 0.353 |
|  |  | Middle-aged | 0.158 | 0.328 |
|  |  | Young | 0.189 | 0.342 |

7. Binomial mixed model for creaky voice occurences

Formula = CREAKY ~ TONE + ACCENT + AGE + (1|TALKER) + (1|SYLLABLE TYPE) + (1|SYLLABLE POSITION)

|  | Estimate | SE | z | p |
| --- | --- | --- | --- | --- |
| (Intercept) | -2.29 | 0.587 | -3.9 | <0.001 |
| TONE1 | -7.02 | 1.016 | -6.91 | <0.001 |
| TONE2 | -4.208 | 0.307 | -13.72 | <0.001 |
| TONE3 | -5.142 | 0.445 | -11.57 | <0.001 |
| TONE5 | -3.971 | 0.309 | -12.86 | <0.001 |
| TONE6 | -3.916 | 0.307 | -12.77 | <0.001 |
| ACCENTHKC | 1.013 | 0.325 | 3.12 | 0.002 |
| AGESenior | -1.441 | 0.48 | -3 | 0.003 |
| AGEYoung | 0.494 | 0.324 | 1.53 | 0.127 |

8. Percentage of creaky voice across tones, accents and age groups

| Tone | Accent | Age | Total # of syllables | Total # of creaky syllables | Percentage |
| --- | --- | --- | --- | --- | --- |
| T1 | GZC | Senior | 88 | 0 | 0.00% |
| T1 | GZC | Middle-aged | 147 | 0 | 0.00% |
| T1 | GZC | Young | 179 | 0 | 0.00% |
| T2 | GZC | Senior | 85 | 0 | 0.00% |
| T2 | GZC | Middle-aged | 152 | 1 | 0.66% |
| T2 | GZC | Young | 176 | 0 | 0.00% |
| T3 | GZC | Senior | 74 | 0 | 0.00% |
| T3 | GZC | Middle-aged | 149 | 0 | 0.00% |
| T3 | GZC | Young | 178 | 0 | 0.00% |
| T4 | GZC | Senior | 74 | 8 | 10.81% |
| T4 | GZC | Middle-aged | 131 | 25 | 19.08% |
| T4 | GZC | Young | 170 | 41 | 24.12% |
| T5 | GZC | Senior | 64 | 0 | 0.00% |
| T5 | GZC | Middle-aged | 119 | 1 | 0.84% |
| T5 | GZC | Young | 156 | 0 | 0.00% |
| T6 | GZC | Senior | 62 | 0 | 0.00% |
| T6 | GZC | Middle-aged | 121 | 0 | 0.00% |
| T6 | GZC | Young | 152 | 1 | 0.66% |
| T1 | HKC | Senior | 118 | 0 | 0.00% |
| T1 | HKC | Middle-aged | 291 | 0 | 0.00% |
| T1 | HKC | Young | 297 | 1 | 0.34% |
| T2 | HKC | Senior | 118 | 1 | 0.85% |
| T2 | HKC | Middle-aged | 294 | 5 | 1.70% |
| T2 | HKC | Young | 296 | 8 | 2.70% |
| T3 | HKC | Senior | 105 | 2 | 1.90% |
| T3 | HKC | Middle-aged | 290 | 1 | 0.34% |
| T3 | HKC | Young | 283 | 3 | 1.06% |
| T4 | HKC | Senior | 120 | 11 | 9.17% |
| T4 | HKC | Middle-aged | 294 | 83 | 28.23% |
| T4 | HKC | Young | 289 | 115 | 39.79% |
| T5 | HKC | Senior | 93 | 1 | 1.08% |
| T5 | HKC | Middle-aged | 211 | 7 | 3.32% |
| T5 | HKC | Young | 264 | 6 | 2.27% |
| T6 | HKC | Senior | 94 | 0 | 0.00% |
| T6 | HKC | Middle-aged | 239 | 6 | 2.51% |
| T6 | HKC | Young | 230 | 8 | 3.48% |

9. Means and SD of CPP for creaky T4 syllables

| ACCENT | Mean | SD |
| --- | --- | --- |
| GZC | -0.2872 | 0.3388 |
| HKC | -0.1623 | 0.3117 |

10. Mixed model for CPP in creaky T4 syllables

FORMULA = CPP ~ ACCENT + (1|TALKER) + (1|SYLLABLE TYPE) + (1|SYLLABLE POSITION)

|  | Estimate | SE | df | *t* | *p* |
| --- | --- | --- | --- | --- | --- |
| (Intercept) | -0.266 | 0.102 | 4.376 | -2.610 | 0.054 |
| ACCENTHKC | 0.107 | 0.044 | 89.794 | 2.460 | 0.016 |

12. Mixed model for H1*-H2* in creaky T4 syllables

FORMULA = H1*-H2* ~ ACCENT + GENDER + (1|TALKER) + (1|SYLLABLE TYPE) + (1|SYLLABLE POSITION)

|  | Estimate | SE | df | *t* | *p* |
| --- | --- | --- | --- | --- | --- |
| (Intercept) | -0.066 | 0.077 | 7.941 | -0.855 | 0.418 |
| ACCENT:HKC | 0.121 | 0.051 | 116.086 | 2.377 | 0.019 |
| GENDER:Male | -0.294 | 0.046 | 106.097 | -6.334 | <0.001 |
